# New Variant of Varicella-Zoster Virus

**DOI:** 10.3201/eid0812.020118

**Published:** 2002-12

**Authors:** Graham A. Tipples, Gwen M. Stephens, Chris Sherlock, Margrit Bowler, Benny Hoy, Darrel Cook, Charles Grose

**Affiliations:** * National Microbiology Laboratory, Winnipeg, Manitoba, Canada; † St. Paul’s Hospital, Vancouver, British Columbia, Canada; ‡ British Columbia Centre for Disease Control, Vancouver, British Columbia, Canada; § University of Iowa, Iowa City, Iowa, USA

**Keywords:** varicella-zoster virus, variant, mutant

## Abstract

In 1998, a varicella-zoster virus glycoprotein E (gE) mutant virus (VZV-MSP) was isolated from a child with chickenpox. VZV-MSP, representing a second VZV serotype, was considered a rarity. We isolated another VZV-MSP-like virus from an elderly man with herpes zoster. These gE mutant viruses may have arisen through independent mutation or may represent a distinct VZV subpopulation that emerged more than 50 years ago.

In 1998, a VZV mutant virus (VZV-MSP) was discovered in Minneapolis-St. Paul, Minnesota; the virus had a missense mutation in the preponderant surface glycoprotein called gE (1,2). The mutation in VZV-MSP led to a lost B-cell epitope in the gE ectodomain. We define “mutant” as a virus with distinctive phenotypic characteristics associated with a nucleotide polymorphism and “variant” as a more inclusive term to include any virus with nucleotide polymorphisms whether or not the virus has a distinguishable associated phenotype. Many methods for differentiating VZV variants and their application to molecular epidemiologic studies have been described (3–8). However, no nucleotide polymorphisms, with the exception of the gE mutation in VZV-MSP, have conclusively been linked to distinguishable phenotypes.

Before 1995, a general assumption was that only one VZV serotype was found around the world (5,9). Similarly, the published sequence of the Dumas laboratory strain was generally accepted as the standard for all strains (10,11). Whether the VZV-MSP mutant virus exhibits increased fitness is not yet known. However, the mutant virus has a recognizable phenotype consisting of accelerated cell spread in both cell culture and the SCID-hu mouse (severe combined immunodeficient mouse with a human skin implant) model of VZV pathogenesis as well as a strikingly different pattern of egress, as documented by scanning electron microscopy (2). VZV-MSP was isolated from a child with leukemia who contracted chickenpox and was admitted to hospital for treatment with intravenous acyclovir. The discovery of a VZV gE variant virus was unexpected; one hypothesis is that the mutation occurred for the first time in the virus replicating in the index case and that the discovery was a serendipitous event unlikely to ever occur again.

We describe the second case of a gE mutant virus found in North America. This discovery provides evidence that the gE mutant virus was unlikely to have occurred as a serendipitous chance mutation.

## Case Report

In December 1999, a 75-year-old man from Vancouver, British Columbia, Canada, arrived at the hospital with severe zoster lesions on his face (in division V1 of the left trigeminal cranial nerve). Painful postherpetic neuralgia with headaches, associated with occasional blurring of vision and lasting for 6 months, developed. The patient was treated with famciclovir (500 mg three times a day for 7 days). A swab of the facial vesicles was obtained from the patient during acute zoster before famciclovir treatment.

Characteristic VZV cytopathogenic effect was seen after 9 days in cell culture. Results of immunofluorescence staining, performed with a commercial kit (Meridian Diagnostics, Cincinnati, OH) by using the anti-gE monoclonal antibody (MAb 3B3), were negative. Staining with monoclonal antibody conjugates for cytomegalovirus and human herpes simplex viruses was also negative. Electron microscopy indicated the presence of enveloped virus characteristic of herpesviruses.

 Polymerase chain reaction amplification and subsequent DNA sequencing confirmed the virus genome to be VZV (hereafter referred to as VZV-BC). The VZV-BC strain was then analyzed in ORF68 (encodes gE and contains the 3B3 MAb epitope) and found to have an identical nucleotide sequence to VZV-MSP (GenBank accession no. AY005330). The VZV-BC and VZV-MSP strains differed from the prototype Dumas VZV strain (GenBank accession no. X04370) in ORF68 by a single G448A nucleotide mutation (D150N amino acid substitution) in the 3B3 epitope of gE (Table). VZV-MSP and VZV-BC were also identical in nucleotide sequence in ORF62 (IE62) and ORF37 (gH). In short, VZV-BC was closely related genetically, if not identical, to the VZV-MSP mutant virus.

**Table:** Amino acid sequences of VZV strains Dumas, MSP and BC in the 3B3 monoclonal antibody epitope^a^

	Codon											
	150	151	152	153	154	155	156	157	158	159	160	161
Dumas	D	Q	R	Q	Y	G	D	V	F	K	G	D
VZV-MSP	N	Q	R	Q	Y	G	D	V	F	K	G	D
VZV-BC	N	Q	R	Q	Y	G	D	V	F	K	G	D

^a^VZV-BC, varicella-zoster virus found in British Columbia; VZV-MSP, varicella-zoster virus glycoprotein E mutant virus.

## Conclusions

VZV-MSP was the first community-acquired VZV strain shown to have a phenotype distinguishable from the traditional VZV phenotype. This case report documents the existence of a second isolate with the same gE genotype. VZV-MSP has been shown to have attributes consistent with increased virulence in the SCID-hu mouse model (2). Both VZV-MSP and VZV-BC are escape mutants (i.e., they have lost a B-cell epitope present in the prototypic Dumas virus from Holland, as well as other viruses from North America, Europe, and Asia, including the Oka varicella vaccine strain from Japan) (11). We have previously shown that the same gE epitope is involved in both complement-dependent neutralization (12) as well as antibody-dependent cellular cytotoxicity (13). Considering the cell culture and animal model data for VZV-MSP and the lost B-cell epitope resulting from the D150N gE mutation, this mutation should be monitored to determine whether it plays a role in more severe cases of varicella or zoster or in breakthrough varicella in previously immunized children.

The origin of this variant may be the same gE mutation, occurring separately in two patients by chance. The argument favoring chance was raised after the discovery of the first gE mutant; however, we find this argument less compelling after the discovery of a second VZV strain with an identically mutated gE antigenic site, just 4 years later. Two other possibilities for its origin seem more plausible. The same gE mutation may have occurred from selective antibody pressure. In an article from the laboratory of the Nobel laureate Zinkernagel (14), the investigators showed that antibody escape variant viruses were more likely to arise in animals with deficient cellular immunity. The original VZV-MSP isolate was obtained from chickenpox in a child under treatment for leukemia, a condition known to be associated with depressed immunity. The second isolate was obtained from an elderly adult with herpes zoster, a condition that may be related to diminished immunity from aging. One of the antibody-escape glycoprotein mutant viruses described by the Zinkernagel laboratory had the identical aspartic acid to asparagine mutation seen in the two VZV-MSP glycoprotein variant viruses. However, mutant viruses probably would have been discovered in the immunocompromised population. The most likely scenario is that VZV-MSP is a previously unrecognized subpopulation of VZV that has been circulating for more than 50 years in circumscribed regions of northern United States and Canada. The timeline is based on the decade when the British Columbia patient likely first contracted chickenpox as a child. The gE sequencing analyses of 30 isolates by Shankar et al. (15) did not uncover a D150N gE mutation; therefore, larger studies need to be conducted to determine the prevalence of this mutant virus in North America.

We conclude that diagnosticians should be aware of the existence of VZV-MSP-like strains and the potential for false-negative testing results with single Mab-based antigen-detection kits for VZV or other herpesviruses. Further clinical observations are needed to fully assess the biological significance of the VZV gE mutant virus.
